# Initial Condition Assessment for Reaction-Diffusion Glioma Growth Models: A Translational MRI-Histology (In)Validation Study

**DOI:** 10.3390/tomography7040055

**Published:** 2021-10-29

**Authors:** Corentin Martens, Laetitia Lebrun, Christine Decaestecker, Thomas Vandamme, Yves-Rémi Van Eycke, Antonin Rovai, Thierry Metens, Olivier Debeir, Serge Goldman, Isabelle Salmon, Gaetan Van Simaeys

**Affiliations:** 1 Department of Nuclear Medicine, Hôpital Erasme, Université Libre de Bruxelles, Route de Lennik 808, 1070 Brussels, Belgium; antonin.rovai@erasme.ulb.ac.be (A.R.); serge.goldman@ulb.be (S.G.); gaetan.vansimaeys@ulb.be (G.V.S.); 2 Center for Microscopy and Molecular Imaging (CMMI), Université Libre de Bruxelles, Rue Adrienne Bolland 8, 6041 Charleroi, Belgium; christine.decaestecker@ulb.be (C.D.); yrvaneycke@gmail.com (Y.-R.V.E.); olivier.debeir@ulb.be (O.D.); isabelle.salmon@erasme.ulb.ac.be (I.S.); 3 Laboratory of Image Synthesis and Analysis (LISA), École Polytechnique de Bruxelles, Université Libre de Bruxelles, Avenue Franklin Roosevelt 50, 1050 Brussels, Belgium; thomas.vandamme@ulb.be (T.V.); thierry.metens@ulb.be (T.M.); 4 Department of Pathology, Hôpital Erasme, Université Libre de Bruxelles, Route de Lennik 808, 1070 Brussels, Belgium; laetitia.lebrun@erasme.ulb.ac.be; 5 Department of Radiology, Hôpital Erasme, Université Libre de Bruxelles, Route de Lennik 808, 1070 Brussels, Belgium

**Keywords:** cellularity, digital pathology, glioma, histology, magnetic resonance imaging, reaction-diffusion model, registration, tumor growth modeling, 3D printing

## Abstract

Reaction-diffusion models have been proposed for decades to capture the growth of gliomas. Nevertheless, these models require an initial condition: the tumor cell density distribution over the whole brain at diagnosis time. Several works have proposed to relate this distribution to abnormalities visible on magnetic resonance imaging (MRI). In this work, we verify these hypotheses by stereotactic histological analysis of a non-operated brain with glioblastoma using a 3D-printed slicer. Cell density maps are computed from histological slides using a deep learning approach. The density maps are then registered to a postmortem MR image and related to an MR-derived geodesic distance map to the tumor core. The relation between the edema outlines visible on T2-FLAIR MRI and the distance to the core is also investigated. Our results suggest that (i) the previously proposed exponential decrease of the tumor cell density with the distance to the core is reasonable but (ii) the edema outlines would not correspond to a cell density iso-contour and (iii) the suggested tumor cell density at these outlines is likely overestimated. These findings highlight the limitations of conventional MRI to derive glioma cell density maps and the need for other initialization methods for reaction-diffusion models to be used in clinical practice.

## 1. Introduction

Gliomas are the most common primary brain tumors. Diffuse gliomas, which include their most aggressive form glioblastoma (GBM), are known to be highly infiltrative [1], with the presence of tumor cells reported as far as 4 cm from the gross tumor [2]. The early diagnosis and follow-up of gliomas usually rely on magnetic resonance imaging (MRI). However, whereas recent advents in MR technologies have given access to a significantly deeper insight into the tumor biology, none of the routinely acquired MR sequences allows to directly assess the whole extent of the tumor cell invasion. Instead, tumor-induced alterations of the microenvironment are seen on MR images, such as peritumor vasogenic edema visible on T2/T2-FLAIR sequences and the enhancing tumor core visible on T1-weighted sequences with injection of gadolinium-based contrast agent (T1Gd). Peritumor vasogenic edema originates from an increase in the blood–brain barrier (BBB) permeability induced by the release of vascular endothelial growth factor (VEGF) by tissues under hypoxic stress [3,4] combined with changes in the brain hydrodynamic pressure [5]. The formation of an enhancing tumor core results from a breakdown of the BBB subsequent to neo-vascularization induced by VEGF, allowing gadolinium-based contrast agents to diffuse freely into brain tissues [3].

Pathological and molecular examination carried out on resected or biopsied tissue samples remains the gold standard method to confirm the diagnosis and determine the histological type and grade [6]. According to the 2016 WHO classification of the central nervous system tumors, the identification of infiltrative patterns is essential in the differential diagnosis of diffuse versus pilocytic astrocytomas, a more-circumscribed neoplasm. An overall assessment of the tumor invasion extent would also be beneficial for improved surgery and radiotherapy planning [7]. However, due to their highly invasive nature and their long processing times, the number and frequency of biopsies are restricted, limiting the use of pathological examination as a proper tool for assessing tumor invasion and highlighting the complementarity of radiological examination [8]. In this matter, quantitatively linking glioma cell invasion patterns observed histologically to MR-visible abnormalities would be of great interest. Such information would allow noninvasive assessment of the glioma extent beyond the visible outlines of the tumor while providing a better interpretation of the observed abnormalities.

Mathematical glioma growth modeling has addressed the problem of estimating glioma cell distribution within brain tissues and predicting its temporal evolution. Among the investigated models, reaction-diffusion models first introduced by Murray and colleagues in the early 1990s [9] are the most widely used, with potential applications for patient follow-up and improved radiotherapy planning [7]. These models rely on a reaction-diffusion equation to capture the spatial-temporal evolution of a tumor cell density function:

{(1)∂c(r, t)∂t=▽·(D(r)▽c(r, t))+ρ c(r, t)(1−c(r, t))∀r∈Ω,∀t>0,(2)c(r, 0)=c0(r)∀r∈Ω,(3)D(r)▽c(r, t)·n∂Ω(r)=0∀r∈∂Ω,

where c(r,t) is the tumor cell density at position r and time *t* normalized by the maximum carrying capacity $c_{\max}$ of brain tissues (i.e., c(r,t)∈[0,1],∀r,t), D(r) is the symmetric tumor cell diffusion tensor at position r, ρ is the tumor cell proliferation rate, $c_{0}\left(r\right)$ is the initial tumor cell density at position r, and $n_{\partial_{\Omega }}\left(r\right)$ is a unit normal vector pointing outwards the boundary $\partial_{\Omega }$ of the brain domain Ω at position $r\in \partial_{\Omega }$. The reaction term appearing in Equation (1) is logistic, which accounts for the (linear) saturation of the tumor cell proliferation due to resource restrictions and ensures a maximum cell density of $c_{max}$ (i.e., a maximum normalized cell density *c* of 1). In the case of a constant and isotropic diffusion coefficient *d*, Equation (1) is also referred to as the Fisher’s equation, whose asymptotic behavior on the infinite cylinder has been extensively studied previously [10,11]. For the sake of completeness, it should, however, be noted that other reaction terms have been proposed previously for reaction diffusion models, namely, exponential ρc(r,t) (no saturation) and Gompertzian $\rho \;c\left(r,t\right)\;\ln\left(1/c(r,t)\right)$ (exponential saturation) [12,13]. Equation (2) specifies the initial condition of the problem. Equation (3) provides no-flux Neumann boundary conditions reflecting the inability of tumor cells to diffuse across $\partial_{\Omega }$.

Reaction-diffusion models as the one in Equations (1)–(3) are particularly attractive for clinical applications as they only have a few parameters that could be assessed from patient imaging data. For instance, based on prior observations that tumor cells rather migrate along than across white matter tracts, Jbabdi and colleagues proposed a method to derive a tumor cell diffusion tensor D(r) from diffusion tensor imaging (DTI) data [14]. For a more detailed overview of reaction-diffusion glioma growth modeling and its potential clinical applications, the reader is referred to the works in [7,9,11,12,15].

One problem arising when attempting to solve Equations (1)–(3) from actual imaging data of newly diagnosed glioma patients is to estimate the initial cell density $c_{0}\left(r\right)$ at every location of the brain domain Ω. To address this issue, early works on glioma growth modeling proposed to relate the MR-visible abnormalities observed at time *t* to the tumor cell density function c(r,t). For example, Swanson and colleagues suggested to model the MR imaging process as a simple cell density threshold function [15]:

(4) $$\begin{array}{cc} I_{T1\mathrm{Gd}}\left(r,t\right) & =\left\{\begin{array}{cc} 1\; & \mathrm{if}\;c\left(r,t\right)\geq c_{\mathrm{enhancing}}\\ 0\; & \mathrm{otherwise} \end{array}\right., \end{array}$$

(5) $$\begin{array}{cc} I_{T2}\left(r,t\right) & =\left\{\begin{array}{cc} 1\; & \mathrm{if}\;c\left(r,t\right)\geq c_{\mathrm{edema}}\\ 0\; & \mathrm{otherwise} \end{array}\right., \end{array}$$

where $I_{T1\mathrm{Gd}}\left(r,t\right)$ and $I_{T2}\left(r,t\right)$ are, respectively, the imaging functions of the T1Gd and T2/T2-FLAIR MR sequences indicating whether the abnormality is visible at location r and time *t* on the sequence, and $c_{\mathrm{enhancing}}$ and $c_{\mathrm{edema}}$ are the corresponding tumor cell density detection thresholds. Based on these assumptions, it was suggested that the outlines of the tumor enhancing core in T1Gd images and of the vasogenic edema in T2/T2-FLAIR images would correspond to iso-contours of the tumor cell density function:

(6) $$c\left(r,t\right)=\left\{\begin{array}{cc} c_{\mathrm{enhancing}}\; & \mathrm{for}\;r\in \partial \Omega_{\mathrm{enhancing}}\\ c_{\mathrm{edema}}\; & \mathrm{for}\;r\in \partial \Omega_{\mathrm{edema}} \end{array}\right.,$$

where $\partial_{\mathrm{enhancing}}$ and $\partial_{\mathrm{edema}}$ are, respectively, the enhancing core and edema outlines. The authors also suggested hypothetical values for $c_{\mathrm{enhancing}}$ and $c_{\mathrm{edema}}$ of 0.80 and 0.16, respectively [15], although no rationale was provided for these values. Building upon this work, Konukoglu and colleagues proposed a fast-marching approach to construct an approximate solution of Equations (1)–(3) at imaging time satisfying Equation (6) [11]. More recently, the same group suggested that—for a spatially constant and isotropic diffusion coefficient $d_{\mathrm{white}}$ and from a certain distance to the tumor core—the tumor cell density in white matter would approximately decrease exponentially with the distance *d* to the core [7]:

(7) $$c\left(r,t\right)\propto \exp\left(-\frac{d(r)}{\lambda_{\mathrm{white}}}\right),$$

where $\lambda_{\mathrm{white}}$ is the infiltration length of tumor cells in white matter given by $\sqrt{d_{\mathrm{white}}/\rho }$. Provided two iso-cell density contours and a distance map to the tumor core, the value of $\lambda_{\mathrm{white}}$ could therefore theoretically be assessed using Equation (7). A similar reasoning had been previously applied in [9] for glioma growth modeling in computed tomography images, leading to the same expression as Equation (7) for the initial condition $c_{0}\left(r\right)$.

Nevertheless, these attempts to derive a tumor cell density distribution from MR images rely on the existence of cell density iso-contours in MR images and are based on unverified assumptions in Equations (4)–(7). However, the extent of vasogenic edema is, for example, known to be impacted by the administration of corticosteroids and anti-angiogenic treatments, independent of the tumor progression [3]. As reaction-diffusion models are highly sensitive to the provided initial tumor cell density distribution $c_{0}\left(r\right)$ [16], the validation of the aforementioned hypotheses is crucial for these models to be usable in clinical routine. In this work, we propose to verify these assumptions, as well as the value of 0.16 suggested for $c_{\mathrm{edema}}$ in [15], through a translational MRI/histology study conducted in a case of non-operated GBM. To this end, stereotactic histological analyses are performed using a 3D-printed slicer designed from antemortem MRI data. Cell density maps are computed automatically from the scanned histological slides using a deep convolutional neural network and related to an MR-derived geodesic distance map to the tumor core. The relation between the edema outlines and the geodesic distance to the core is also investigated. Our results highlight the limitations of using routine MRI to derive glioma cell density maps and point out the need for other validated initialization methods to make reaction-diffusion growth models usable in clinical practice.

## 2. Materials and Methods

### 2.1. Clinical Case

For the needs of this work, the case of a deceased 89-year-old female patient with GBM was studied retrospectively. The patient underwent an MRI examination in November 2017 in the context of a clinical frontal syndrome, which revealed a massive right-frontal expanding lesion with intense heterogeneous enhancement post-injection of gadolinium contrast agent and a necrotic core, surrounded by a large area of perilesional edema. Considering the patient’s age, a consensual decision was taken not to perform surgery and a diagnosis of GBM was made exclusively based on MRI. A corticotherapy (methylprednisolone) and a palliative chemotherapy-based treatment (temozolomide, TMZ) were initiated right after diagnosis. In December 2017, after one cycle of TMZ, the patient died of a septic shock caused by bowel perforation. An autopsy was carried out within 12 h after death, as part of which the patient’s brain was collected and fixed in formalin for 24 months. Upon microscopic examination, an infiltrating glioma with high cellularity, astrocytic phenotype cells, and nuclear atypia was observed along with glomeruloid vascular proliferation and areas of pseudo-palissading necrosis.

### 2.2. MR Image Acquisitions

The T1 (TR = 8 ms, TE = 2.9 ms, TI = 950 ms, FA = 8°) and T2-FLAIR (TR = 4800 ms, TE = 320 ms, TI = 1650 ms) MR images routinely acquired at diagnosis time on a 3T Achieva scanner (Philips Healthcare, Best, The Netherlands)—referred to as the ‘in vivo’ images hereafter—were retrospectively used in this work for registration guidance and delineation of the vasogenic edema.

Additionally, a T1 BRAVO ‘ex vivo’ acquisition (TR = 8.264 ms, TE = 3.164 ms, TI = 450 ms, FA = 12°) of the brain placed inside the 3D-printed slicer (see below) was performed on a 3T Signa PET/MR scanner (GE Healthcare, Chicago, IL, USA) right before slicing. It should, however, be noted that brain fixation has caused convergence of the T1 values of white and gray matter, as reported in [17,18]. Consequently, the acquired ex vivo T1 image rather has a proton-density (PD) contrast. Furthermore, note that drainage of the extracellular fluid made it impossible to delineate edema regions on postmortem T2-FLAIR images, motivating the use of a registered antemortem T2-FLAIR image for edema delineation hereafter.

### 2.3. Slicer Design and Tissue Sampling

**Figure 1:**
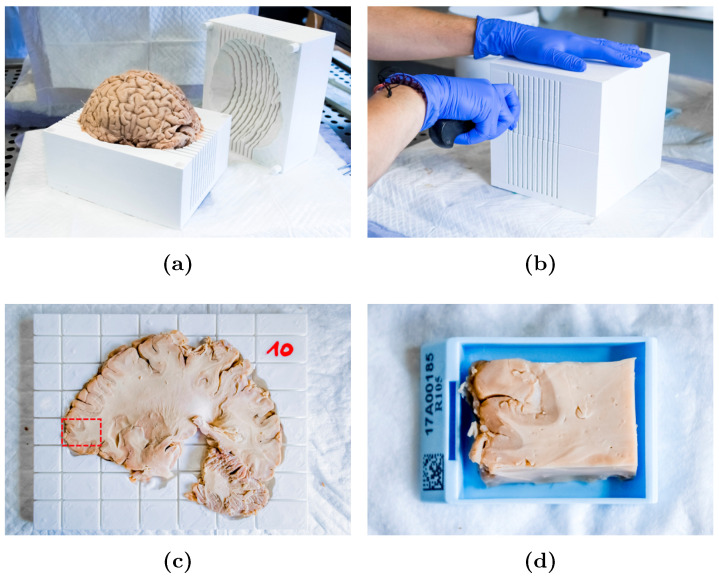
Brain slicing and sample collection procedure. (**a**) The brain is placed inside the 3D-printed slicer. (**b**) Sagittal slices are cut carefully. (**c**) Each brain slice is placed inside its cutting guide. (**d**) Sample blocks are cut with a scalpel along the grooves and placed into standard cassettes.

**Figure A1:**
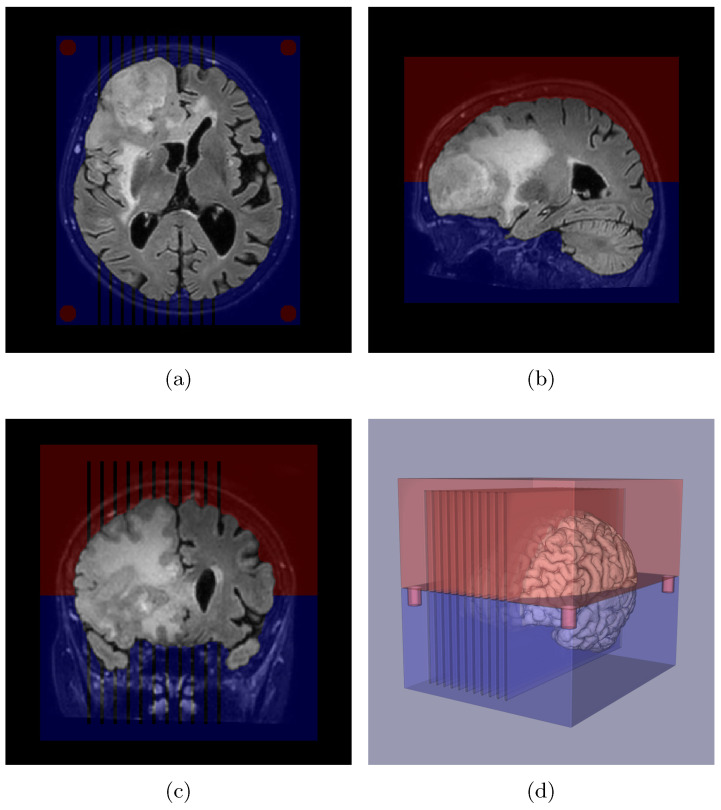
Brain slicer design superimposed to the in vivo T2-FLAIR image used as template in axial (**a**), sagittal (**b**), coronal (**c**) and 3D (**d**) views.

To relate histological observations to the abnormalities in MR images and to the MR-derived distance map to the tumor core (see below), a brain slicer was designed based on the in vivo T2-FLAIR image, then 3D printed. Such a slicer allows the brain to be repositioned in antemortem imaging orientation and facilitates the cutting of sagittal brain slices. The slicer design procedure is illustrated in Figure A1. A similar slicer design approach was previously adopted in [19]. Ten guides were also designed to ease the collection of sample blocks from brain slices that are compatible with our histological processing chain. These consist of plates with grooves from which the brain slice volume was subtracted. The brain slicing and samples collection procedure is illustrated in Figure 1. More details on the design steps of the slicer and of the cutting guides are available in Appendix A.

### 2.4. Sample Processing and Analysis

Twenty-eight tissue samples were selected within the tumor core, as well as within and beyond the peritumoral edematous region based on the in vivo T2-FLAIR image. The samples were formalin-fixed and paraffin-embedded (ISO 15189). 5 μm slides were cut from each sample and stained with hematoxylin and eosin (HE). The 28 stained slides were scanned in 20× mode (per-mode=symbol 0.46 μm/px) on a calibrated NanoZoomer 2.0-HT digital slide scanner (Hamamatsu Photonics, Japan) for numerical processing. The stained slides were independently examined by an experienced pathologist blinded to MRI for the presence of pseudo-palisading necrosis, tumor cells (in block or infiltrating), glomeruloid vascular proliferation, and edema. As will be further discussed, immunohistochemistry (IHC) staining was also investigated but did not provide satisfactory results due to over-fixation of the brain tissues.

### 2.5. Cell Density Maps

**Figure 2:**
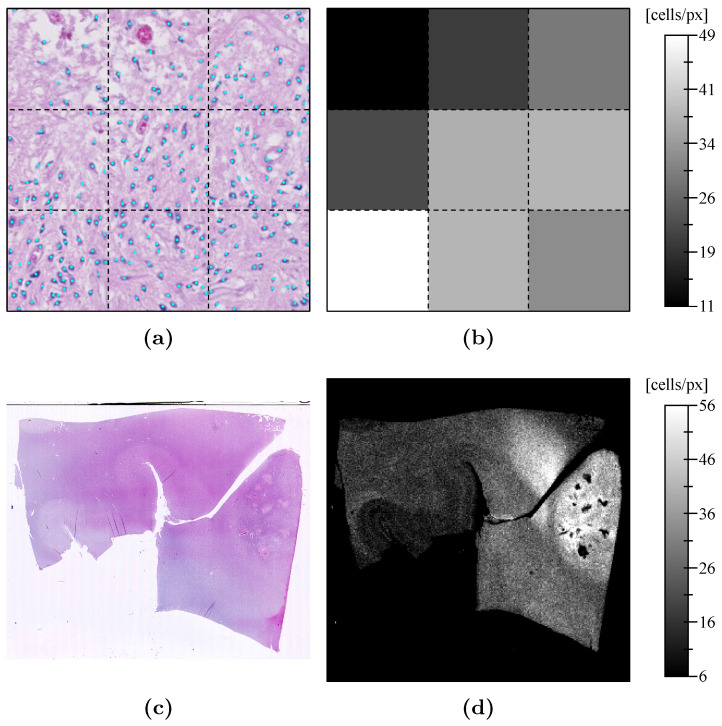
Cell density map computation procedure. (**a**) 3×3 adjacent tiles (dotted squares) with dimensions 100 px×100 px and pixel size 1 μm×1 μm extracted from the resampled slide in panel (**c**). Cell nuclei detected by the deep convolutional neural network are indicated with cyan dots. (**b**) Corresponding 3×3 pixels (dotted squares) of the cell density map with pixel size 0.1 mm×0.1 mm whose value is given by the corresponding tile cell count divided by the true tissue area. (**c**) Whole hematoxylin and eosin stained slide (slide 13, see Table A1). (**d**) Corresponding whole computed cell density map.

Cell density maps were computed from the scanned HE slides to highlight tumor cell invasion in normal brain tissues. Each scanned slide was first resampled to an isotropic pixel size of 1 μm×1 μm and divided into adjacent tiles of 100 px×100 px. Cell nuclei within each tile were automatically counted using a weakly-supervised deep learning approach detailed in Appendix B. The counting result was divided by the actual tissue area within the tile, defined as the number of tissue pixels (see Appendix B) times the pixel area ( $10^{-6}$ m$m^{2}$). The computed cell density values were finally stored as a 2D image with a pixel size of 0.1 mm×0.1 mm where each pixel exactly corresponds to one of the 100 px×100 px tiles of the resampled slide. The cell density map computation procedure is illustrated in Figure 2.

In addition, volume cell density values were extrapolated from the computed surface cell density values, as the former are the actual values of interest for the reaction-diffusion growth model in Equations (1)–(3). Under the assumptions that:

1. cell nuclei are approximately spherical;
2. cell nuclei distribution is locally isotropic;
3. tile dimensions are sufficiently large to contain multiple cells;
4. tile dimensions are sufficiently small for the cell density to be considered homogeneous and isotropic;

and denominating *l* the side length of the square tile, a volume cell density value $c_{\mathrm{volume}}$ can be extrapolated for a cube with the same side length *l* from the surface cell density $c_{\mathrm{surface}}$ by

(8) $$c_{\mathrm{volume}}={\left.c_{\mathrm{surface}}\right.}^{\frac{3}{2}}.$$

### 2.6. Cell Density Maps to Ex Vivo T1 Registration

Substantial deformations of the brain occurred between the antemortem MR acquisitions and the postmortem histological analyses. The ex vivo T1 image space was thus used as the reference space for the analyses and the cell density maps were registered to the ex vivo T1 image as follows. The ex vivo T1 image was first resampled by linear interpolation to an isotropic voxel size of 0.5 mm. The 2D cell density maps were artificially extended to 3D images with a thickness of 0.5 mm and resliced to sagittal orientation. The density maps were finally rigidly registered to the corresponding slice of the resampled ex vivo T1 image based on user-defined landmark pairs using an in-house software in C++ based on VTK [20] and ITK [21].

The cell density map registration process was greatly facilitated by the use of a 3D-printed slicer as it allowed to impose the brain slicing orientation. The complex histology slide to MR image registration process in 3D was thus reduced to a simpler MR slice selection followed by the identification of at least 3 landmark pairs in-plane. Furthermore, the computed cell density maps have the great advantage of providing spatial tissue information at an intermediate scale between histological and radiological images, with a contrast similar to T1-weighted MR images. The cell densities of white and gray matter are indeed substantially different—as is their T1 and PD values, which eased the identification of landmarks pairs.

### 2.7. Edema Delineation

To verify the assumptions in Equations (5) and (6), the edema region has to be delineated in the reference ex vivo T1 image space. However, as previously mentioned, the drainage of the extracellular fluid made it impossible to discern vasogenic edema on the ex vivo MR images. The in vivo T2-FLAIR image was thus registered to the reference ex vivo T1 image. To this end, the in vivo T1 image acquired on the same day was first registered on the ex vivo T1 image using rigid followed by B-spline transforms available as part of the Elastix software [22]. The computed transforms were then successively applied to the in vivo T2-FLAIR image. The Elastix parameter files used for registration are available in Appendix C. The edema segmentation was finally performed semi-automatically on the registered T2-FLAIR image using a combination of thresholding and morphological operations.

### 2.8. Distance Map

To verify the assumption in Equation (7), a 3D geodesic distance map to the tumor core across white matter was computed from the ex vivo T1 image. White matter was first segmented using an in-house gradient-based anisotropic diffusion algorithm followed by manual corrections to ensure that no physically incompatible bypass exists between white matter regions. The tumor core was then segmented on the same image using a combination of thresholding and morphological operations. A distance map to the tumor core across the segmented white matter region was finally computed using an adapted implementation of the anisotropic fast marching (AFM) algorithm presented in [23]. Note that as no DTI images were available for the patient, the anisotropy of glioma cell diffusion mentioned above could not be taken into account in this work. A unit isotropic metric tensor field was thus provided to the AFM algorithm, hence the abusive use of the term ‘distance map’ to designate the ‘traveling time map’ returned by the algorithm.

The relation between the edema extent and the geodesic distance map was also investigated using the Hausdorff distance and the average symmetric surface distance (ASSD), computed between the edema outlines and the contours of the binary region obtained by thresholding the distance map. The Hausdorff distance $d_{\mathrm{Hausdorff}}$ and the ASSD $d_{\mathrm{ASSD}}$ between two sets *A* and *B* are, respectively, given by [24]

(9) $$\begin{array}{cc} d_{\mathrm{Hausdorff}}\left(A,B\right) & =\max\left\{\max_{b\in B}\left\{\min_{a\in A}d\left(a,b\right)\right\},\max_{a\in A}\left\{\min_{b\in B}d\left(a,b\right)\right\}\right\}, \end{array}$$

(10) $$\begin{array}{cc} d_{\mathrm{ASSD}}\left(A,B\right) & =\frac{1}{\left|A|+|B\right|}\left(\sum_{b\in B}\min_{a\in A}d\left(a,b\right)+\sum_{a\in A}\min_{b\in B}d\left(a,b\right)\right), \end{array}$$

where d(x,y) is the Euclidian distance between elements *x* and *y*, and |X| is the cardinal of set *X*.

### 2.9. Cell Density Model

As mentioned, the over-fixation of brain tissues prevented any IHC staining. Therefore, HE staining had to be used instead, making it difficult to distinguish infiltrating tumor cells from healthy brain cells on the scanned slides. Consequently, nuclei-based cell density maps were computed, which reflect the total cell density without distinction between tumor and healthy cells. To address this problem, we propose to verify the following equation instead of Equation (7):

(11) $$\begin{array}{cc} c_{\mathrm{total}}\left(r\right) & =c_{\mathrm{tumor}}\left(r\right)+c_{\mathrm{white}}, \end{array}$$

(12) $$\begin{array}{cc} \; & =c_{\mathrm{core}}\exp\left(-\frac{d(r)}{\lambda_{\mathrm{white}}}\right)+c_{\mathrm{white}}, \end{array}$$

where $c_{\mathrm{total}}$, $c_{\mathrm{tumor}}$, and $c_{\mathrm{white}}$ are, respectively, the total, tumor, and healthy cell densities in white matter, and $c_{\mathrm{core}}$ is the tumor cell density iso-value along the tumor core boundary. In this formulation, the cellularity of healthy white matter is supposed to be approximately constant and the invading tumor cells are assumed to be superimposed to a white matter baseline cellularity $c_{\mathrm{white}}$.

To relate the total cell density and the geodesic distance to the tumor core, the registered cell density maps were resampled to the same voxel size as the geodesic distance map (0.5 mm×0.5 mm×0.5 mm) and all available pairs of density/distance values among the segmented white matter voxels were extracted. The values of $c_{\mathrm{core}}$, $\lambda_{\mathrm{white}}$, and $c_{\mathrm{white}}$ in Equation (12) were finally least-squares fitted to the available experimental density/distance pairs using SciPy’s ‘optimize’ module in Python [25].

## 3. Results

**Figure 3:**
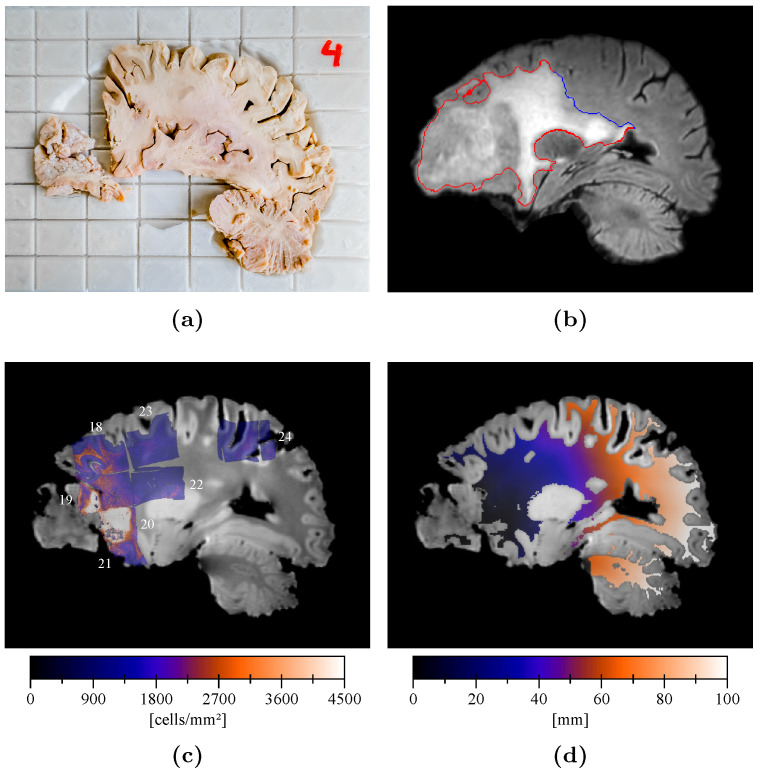
Cell density profile analysis. (**a**) Brain slice inside its 3D-printed cutting guide. (**b**) Corresponding slice of the registered in vivo T2-FLAIR image with segmented edema outlines. The blue and red segments of the outline respectively correspond to free and non-free to diffuse parts of the edema boundary (see Section 4). (**c**) Corresponding slice of the ex vivo T1 image (grayscale) and superimposed registered cell density maps (colored) with their slide number (see Table A1). (**d**) Corresponding slice of the geodesic distance map to the tumor core across white matter.

Figure 3 depicts an example of brain slice in its cutting guide (Figure 3a) with the corresponding registered in vivo T2-FLAIR image slice (Figure 3b), registered cell density maps (Figure 3c), and geodesic distance map (Figure 3d). An over-cellularity front is visible in Figure 3c, progressing from the frontal necrotic tumor core but rapidly decreasing to reach an apparently normal cellularity of around 1450 cell/m$m^{2}$ beyond a geodesic distance of 20 m$m^{2}$ (Figure 3d). The edema outlines, on the other hand, extend to over 50 mm on the depicted image slice (see red and blue delineations in Figure 3b). The distinction between the red and blue segments of the edema outlines in Figure 3b will be used in the discussion.

**Figure 4:**
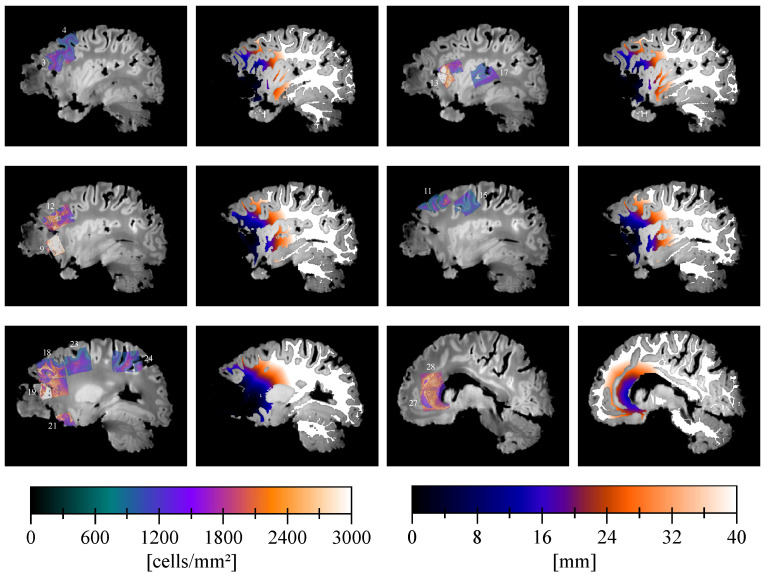
Example of registered cell density maps with their slide number (see Table A1) (**1st and 3rd columns**) and corresponding slices of the geodesic distance map to the tumor core (**2nd and 4th columns**) superimposed to the ex vivo T1 image.

More examples of registered cell density maps with the corresponding slice of the geodesic distance map are depicted in Figure 4. The decreasing behavior of the tumor cell density with the distance to the tumor core was observed among all these examples.

**Figure 5:**
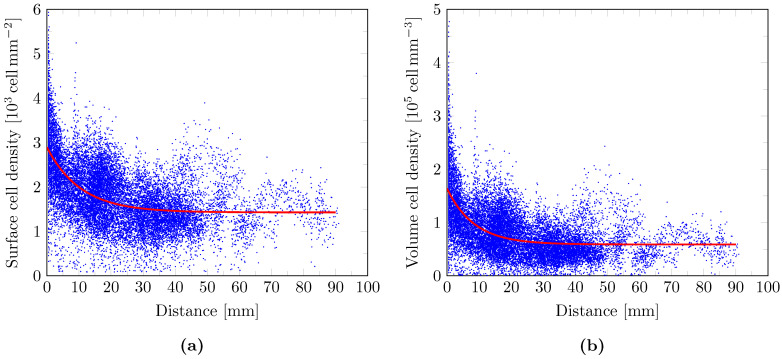
Scatter plot of the surface cell density (**a**) and the extrapolated volume cell density (**b**) versus distance for each available value pairs among white matter voxels (blue dots) with superimposed fitted model curves (red curves).

The available pairs of density/distance values among all white matter voxels are plotted in Figure 5 for the surface density data (Figure 5a) and the volume density data extrapolated using Equation (8) (Figure 5b). The fitted model curve given by Equation (12) is superimposed in red for each plot and the corresponding parameter values are respectively provided in Table 1 and Table 2.

**Figure 6:**
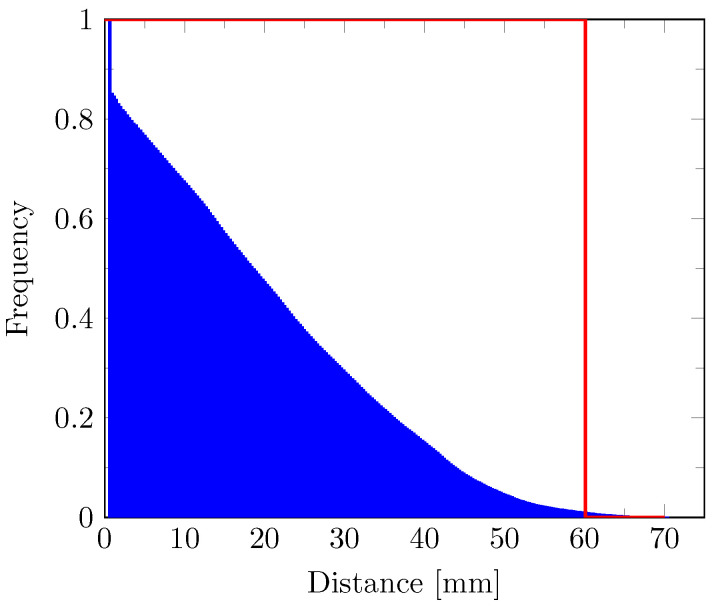
Inverse cumulative distribution of the geodesic distance values along the edema outlines. The expected distribution under the hypothesis of iso-distance edema outlines is plotted in red.

The inverse cumulative distribution of the geodesic distance values along the edema outlines—i.e., the fraction of edema boundary voxels located at a geodesic distance greater or equal to a given value on the *x*-axis—is depicted in Figure 6 (blue). The distribution is rather continuous, suggesting that the edema boundary does not correspond to an iso-distance contour in contrast to the expected step-like distribution superimposed in red in Figure 6. Five percent of the edema boundary voxels are located at a distance greater or equal to 49.4 mm and only 1% are located at a distance greater than 60.1 mm from the tumor core.

**Figure 7:**
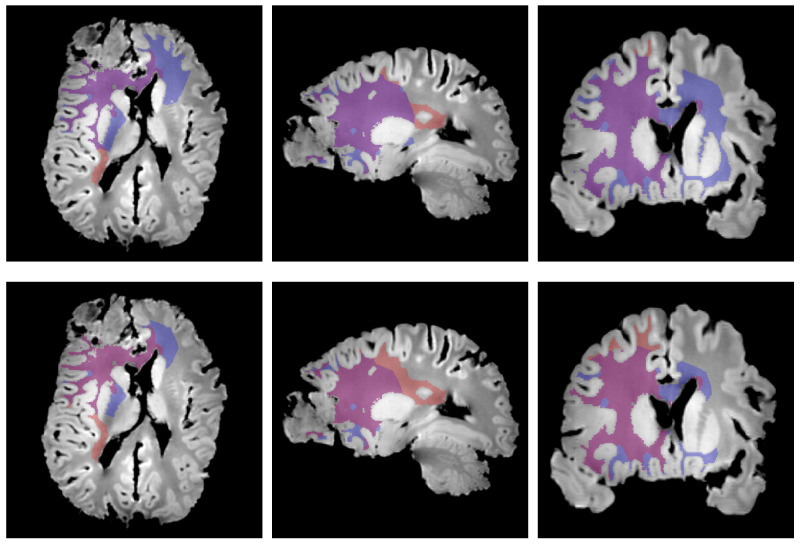
Edema region (red) with superimposed thresholded region of the distance map whose contour minimizes the Hausdorff distance (blue, **1st row**) and average symmetric surface distance (blue, **2nd row**) to the edema contour in axial (**1st column**), sagittal (**2nd column**), and coronal (**3rd column**) planes.

The distance threshold values which provided the smallest Hausdorff distance (24.65 mm) and ASSD (1.97 mm) between the edema outlines and the contour of the thresholded distance map region were 43.5 mm and 35.5 mm, respectively. The corresponding circumscribed volumes are depicted in Figure 7.

The results of the blinded pathological examination and numerical tile processing are summarized in Table A1, reporting the presence of pseudo-palisading necrosis, tumor cells (tumor block versus infiltrative cells), and glomeruloid vascular proliferation, along with the minimum, maximum, and mean cell density and distance values within the corresponding slide. The furthest distance at which suspected infiltrating tumor cells were identified was around 46 mm (slide 7), whereas edema was detected as far as 61.7 mm on the same slide.

## 4. Discussion

The exponentially decreasing glioma cell density profile with distance to the tumor core suggested in [7,9] is compatible with our experimental data, as observed in Figure 5 for both surface (a) and extrapolated volume (b) cell densities. Besides, the fitted value of $\lambda_{\mathrm{white}}$ (8.46 mm) for the volume cell density data is in the same order of magnitude as the one used in [7] for simulations (4.2 mm). The baseline volume cell density value of $0.59\times 10^{5}$ cell m$m^{-3}$ in white matter is also in accordance with the literature, although large variations are observed between the reported values [26]. However, high variance was observed in our experimental data (see the blue point distribution in Figure 5a,b), imputed to errors accumulated throughout the numerous steps of our data processing pipeline. As a result, uncertainties on the fitted parameters are to be expected. In particular, the reported values of $c_{\mathrm{core}}$ in Table 1 and Table 2 were likely underestimated as cell density values of up to $6.1\times 10^{3}$ cell m$m^{-2}$ and $4.8\times 10^{5}$ cell m$m^{-3}$ were, respectively, observed in our surface and volume cell density data (see Figure 5a,b). Consequently, the corresponding fitted values of $\lambda_{\mathrm{white}}$ were likely overestimated.

In contrast, the assumption of iso-cell density edema outlines in Equation (6) was not verified by our experimental data. Indeed, considering a monotonically (exponentially) decreasing relation between the tumor cell density and the distance to the tumor core, iso-density contours should coincide with iso-distance contours. However, our results suggest that edema outlines do not coincide with an iso-distance contour (see Figure 6) and would therefore not correspond to a cell density iso-contour either. This apparent incompatibility of assumptions in Equations (5) and (6) can be explained by the thresholding behavior of the imaging function proposed in Equation (5), from which assumption in Equation (6) was deduced in [15]. In fact, thresholding a spatial function gives rise to iso-value contours only if the function is sufficiently smooth and continuous. In contrast, the tumor cell density function, discretized over the image voxel grid, has many discontinuities at interfaces between white and gray matter and along the brain domain boundaries. Indeed, the difference in tumor cell diffusivity between white and gray matter [7,11,12] gives rise to steep tumor cell gradients at the white/gray matter interfaces, resulting in substantial discontinuities of the cell density function at the voxel level. Along the brain domain boundary, discontinuities are even more pronounced since no tumor cells are allowed to diffuse outside the brain domain (see Equation (3)), resulting in an accumulation of tumor cells. Consequently, edema outlines may not correspond to a cell density iso-contour even if the proposed thresholding behavior of the edema imaging function in Equation (5) turned out to be valid. As an illustration, the edema outlines in Figure 3b were split into blue and red segments, respectively corresponding to parts of the edema boundary where tumor cell diffusion is free (blue) and where diffusion is restricted due to a local decrease in tumor cell diffusivity or the presence of brain boundary (red). From Figure 6, it can be reasonably assumed—taking a margin of 1% for registration errors—that the edema extended up to 60.1 mm from the tumor core, which would correspond to the actual maximum edema extent. Finally, note that, due to the limited number of available cell density map voxels located on the edema outlines, the assumption in Equation (6) could not be directly verified, which motivated the indirect distance-based reasoning herein.

For reasons mentioned above, the thresholding behavior of the edema imaging function proposed in Equation (5) may still be valid even after invalidation of Equation (6). Nevertheless, the thresholded distance map regions whose contour respectively minimizes the Hausdorff distance and the ASSD to the edema outlines were not found to accurately coincide with the edema region, as depicted in Figure 7. Both thresholded distance map regions (blue) in Figure 7 were indeed found to extend farther in the contralateral hemisphere via the corpus callosum and less far towards the right posterior region, compared to the edema region (red). Still, under the hypothesis of a monotonically decreasing relation between the tumor cell density and the distance to the tumor core, the threshold-like imaging function in Equation (5) is therefore incompatible with our results. It should, however, be noted that this apparent inadequacy could still result from residual registration errors between in vivo and ex vivo images. Besides, the use of an isotropic metric tensor for the geodesic map computation instead of a DTI-derived anisotropic metric tensor may also have impacted the presented results. A DTI-derived anisotropic metric tensor would indeed have allowed to account for the well-established preferential migration of glioma cells along white matter tracts [27], originating from both mechanical [28] and molecular [29] factors. Registration of a DTI atlas may have been used to circumvent the lack of DTI data in this study. However, the substantial deformations of the brain observed ex vivo would have required the use of a deformable registration method. Whereas rigid or affine transforms of tensor fields can be trivially performed using matrix products, B-spline transforms of such fields are substantially more challenging as the coordinates system in which the tensor components are expressed is changed locally [30]. One solution could have been to approximate the B-spline transform by an affine transform at every voxel, as suggested in [30], but such developments were out of the scope of this study. We nevertheless investigated the potential impact of an anisotropic metric tensor on the computed distance—or rather ‘arrival time’—maps based on DTI acquisitions of a healthy volunteer. The results presented in Appendix E suggest even shorter arrival times in the contralateral hemisphere due to the high anisotropy in the corpus callosum region, which is not in favor of a coincidence between the edema outlines and a thresholded arrival time map contour (see Figure 7).

The iso-density value of 16% of the maximum cell carrying capacity suggested for the edema contours in [15] was not supported by our experimental data either. Indeed, assuming that Equation (6) would still be verified on the free-to-diffuse part of the edema boundary (blue contour in Figure 3b), the proposed cell density model in Equation (12) with the parameter values fitted to volume cell density data in Table 2 suggests an over-cellularity of only $8.6\times 10^{1}$ cell m$m^{-3}$ with regard to the white matter cellularity baseline $c_{\mathrm{white}}$ at a distance of 60.1 mm from the tumor core—corresponding to the maximum edema extent. This over-cellularity corresponds to only 0.08% of $c_{\mathrm{core}}\leq c_{\max}$, which is far below the commonly accepted value of 16%. In addition, as the value of $c_{\mathrm{core}}$ in Table 2 was likely underestimated as mentioned above, an even lower relative value of $c_{\mathrm{edema}}$ is to be expected. These results are confirmed by blinded pathological examination, which did not reveal any noticeable invasion of the brain parenchyma—even within the edematous region—beyond a distance of 46 mm to the tumor core. This overall analysis does however not exclude the possible presence of isolated infiltrating tumor cells at the edema boundary and beyond as observed in [2,31,32].

Although MRI provides high contrast in soft tissues and is the current standard of care for radiological examination of gliomas, abnormalities visible on conventional MR sequences are not trivially related to the tumor cell invasion extent. A striking illustration of this limitation is the administration of corticosteroid or anti-angiogenic therapies to reduce edema-related symptoms in glioma patients, which does however not stop tumor progression. Consequently, a decoupling arises between tumor progression and its visible effects on the surrounding environment, potentially leading to a misclassification of the disease as responding to treatment. The impact of such therapies on the MR-based follow-up of gliomas has been extensively studied through numerical simulations in [3]. In this work, we invalidated two commonly made assumptions relating the outlines of visible abnormalities on MRI to the tumor cell density function: (i) assuming a threshold-like imaging function for the vasogenic edema in T2/T2-FLAIR images (see Equation (5)), the edema outlines may not correspond to an iso-contour of the cell density function as soon as the migration of tumor cells is locally restricted or prevented, and (ii) at a distance corresponding to the maximum extension of the vasogenic edema, the over-cellularity was found to be negligible in our studied case, as opposed to the previously hypothesized value of 16% of the maximum carrying capacity. These results raise the question of the applicability of the previously proposed methods for deriving glioma cell density distributions from routine MR images referenced in the introduction. Indeed, whereas the method proposed in [11] allows to compute an accurate approximate solution of Equation (1), it still relies on the assumption that iso-density contours can be derived from MR data. In the case of a more simple exponentially decreasing model as in Equation (7) [7,9], iso-density contours would still be required to assess the infiltration length parameter $\lambda_{\mathrm{white}}$. As a high sensibility of the reaction-diffusion tumor growth models to their initial condition was previously reported by our group [16], deriving an initial spatial cell density distribution from medical imaging data that is as reliable as possible is crucial for the model to be applied in clinical practice.

Beyond the field of reaction-diffusion tumor growth modeling, assessing the tumor cell density distribution in gliomas is also of utmost importance for treatment planning and response assessment [33,34,35,36]. To this extend, advanced MR acquisition and processing methods have been proposed previously. In [37], a linear relation was derived voxel-wise between the cell density and the average diffusion coefficient (ADC) value assessed by diffusion-weighted (DW) MRI. ADC maps were then used to assess the parameters and validate a simple logistic tumor growth model in rats with inoculated GBM cells. This approach was more recently used in patients to personalize a reaction-diffusion glioma growth model including the effects of chemoradiation [38]. Furthermore, microstructure imaging using advanced DW-MRI models could provide finer information on the tumor cell density [34,39] but also on the spatial distribution of tumor cells within a voxel [40]. However, this technique would require higher signal-to-noise ratios than achievable on clinical scanners to be used in practice [39]. In [33], glioma invasion is assessed using choline/N-acetylaspartate ratio maps derived from MR spectroscopy and showed promising relations with histology. In [36], anatomic, DW, and dynamic contrast-enhanced MR sequences are used to predict cell density values assessed by biopsies using a random forest classifier, with moderate-to-strong correlations reported between the observed and predicted density values. A similar approach was adopted in [35], with a reported root mean squared error of 1015 cell m$m^{-2}$ on test data. Aside from MRI, the use of positron emission tomography (PET) imaging to assess glioma cell density has also been investigated previously. In [41], a linear relation was shown between the tumor cell density and the uptake of [^18^F]fluoroethyl-L-tyrosine and used in [42] along with conventional MRI to personalize a reaction-diffusion tumor growth model. Finally, combined [^11^C]methionine and [^18^F]fluorodeoxyglucose PET imaging was used in [43] to improve the detection of glioma cell infiltration. As can be noticed, many imaging techniques have been proposed to assess glioma cell density distributions in vivo but no consensual clinical procedure has seemed to emerge so far, leaving the question open.

This study was however prone to several limitations. First, due to the scarcity of the human body material analyzed—a non-operated brain with GBM, this study was based on a single case and should be further carried out on a larger diffuse glioma cohort of various grades. In addition, the use of murine glioma models for conducting such studies at a larger scale would also be of interest but is restricted due to reported substantial differences in cortical [44], glial [45], and endothelial [46] cells between human and mouse brain. Consequently, murine glioma models are known to only partially reflect the characteristics of human gliomas [47]. Second, IHC staining could not be performed on the autopsied material, which has prevented the specific identification of tumor cells. Instead, HE staining was used in this work and the overall cellularity was analyzed. The assumption was made that the cellularity baseline was approximately constant across healthy white matter and that the over-cellularity observed locally was exclusively attributed to tumor cell invasion—which seems reasonable as tumor-induced recruitment of inflammatory cells is limited in brain tissues. As a consequence, the identification of isolated infiltrating tumor cells on pathological examination may have been prevented. Third, the substantial deformation of the brain between in vivo and ex vivo imaging—with a volume decrease estimated to 16% ex vivo based on MRI—may have resulted in partial distortion of the ex vivo MR-derived distance map compared to in vivo, not entirely compensated by deformable registration of the in vivo edema outlines. Fourth, the effect of the palliative TMZ treatment administered to the patient over the month between the in vivo MRI acquisition and the patient’s death could not be properly assessed as no further medical imaging acquisition was performed after the treatment start. Nevertheless, only limited effects would be expected as the patient underwent a single cycle of TMZ, whereas at least six cycles are classically recommended for GBMs [6,48]. Indeed, although a case of complete response after only one cycle of TMZ has been reported in [49], this case was still presented by the authors as unusual. Besides, the response to TMZ is highly dependent on the MGMT methylation status of the tumor [6,48,49], which could not be determined on this autopsied material. This latter limitation well reflects the difficulties encountered to clinically validate tumor growth models in typically multi-treated glioma patients.

We would finally like to emphasize that the automated cell density map computation and histological slide to MR image registration procedures described in this work are not limited to the problem addressed herein and could be applied to various histological stainings, imaging modalities, and organs, such as prostate. Besides, the use of tailor-made 3D-printed slicer and cutting guides makes it possible to precisely analyze whole organ slices at low cost even for centers that do not have access to whole organ slice microscopy, opening tremendous possibilities for translational microscopic/macroscopic imaging research [50].

## 5. Conclusions

Through a translational radiological/histological analysis performed on a case of non-operated glioblastoma, we invalidated two commonly made assumptions relating the outlines of the visible abnormalities in magnetic resonance images to the tumor cell density function in the context of reaction-diffusion glioma growth modeling. We showed that, due to local restrictions of the tumor cell migration at brain tissue interfaces and along the brain boundary, the outlines of vasogenic edema in T2-FLAIR images do not generally coincide with a cell density iso-contour, contrary to what was suggested previously. We also found that the commonly adopted tumor cell density iso-value at the edema outlines is likely overestimated as the over-cellularity assessed at the maximum edema extent was found to be negligible in our studied case. This, however, does not exclude the possible presence of isolated tumor cells migrating beyond edema outlines, as previously reported. This work highlights the limitations of using conventional magnetic resonance images to derive tumor cell density maps and points out the need of validating other methods to accurately initialize reaction-diffusion tumor growth models for clinical applications.

## Figures and Tables

**Table 1:** Least-squares fitted values of the cell density model parameters in Equation (12) for the surface cell density data plotted in Figure 5a.

$c_{\mathrm{core}}$ [$10^{3}$ cell m$m^{-2}$]	$\lambda_{\mathrm{white}}$ [mm]	$c_{\mathrm{white}}$ [$10^{3}$ cell m$m^{-2}$]
1.47	10.55	1.43

**Table 2:** Least-squares fitted values of the cell density model parameters in Equation (12) for the volume cell density data extrapolated using Equation (8) and plotted in Figure 5b.

$c_{\mathrm{core}}$ [$10^{5}$ cell m$m^{-3}$]	$\lambda_{\mathrm{white}}$ [mm]	$c_{\mathrm{white}}$ [$10^{5}$ cell m$m^{-3}$]
1.05	8.46	0.59

**Figure A2:**
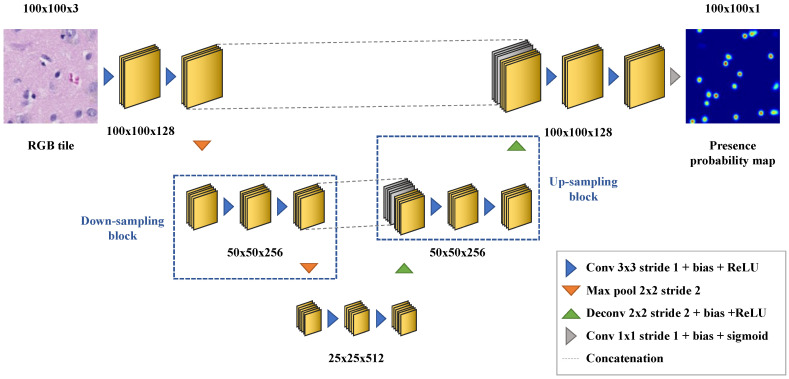
U-Net architecture with its feature map dimensions used for nuclei detection. The network takes 100 px×100 px RGB tiles with pixel size 1 μm×1 μm as input and predicts a nuclei presence probability map with the same spatial dimensions.

**Listing A1:**
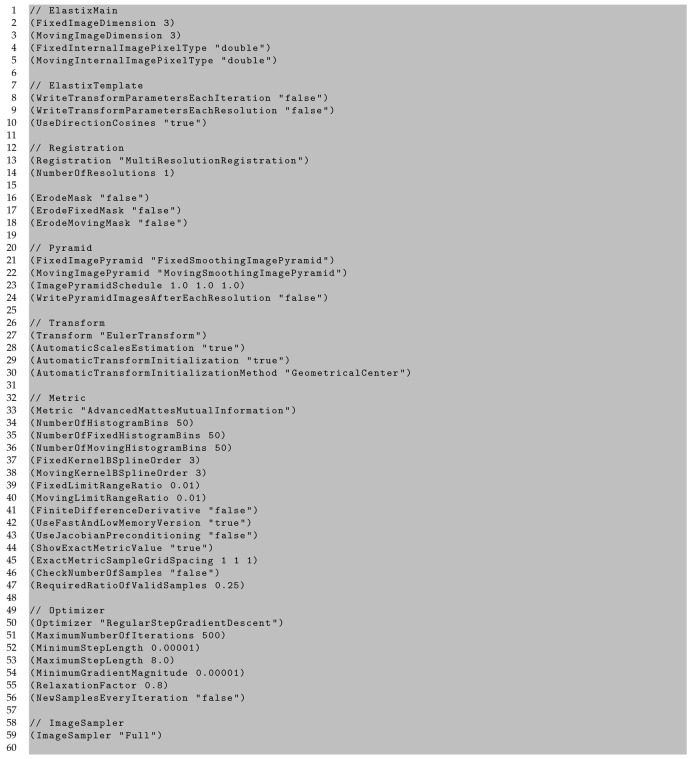

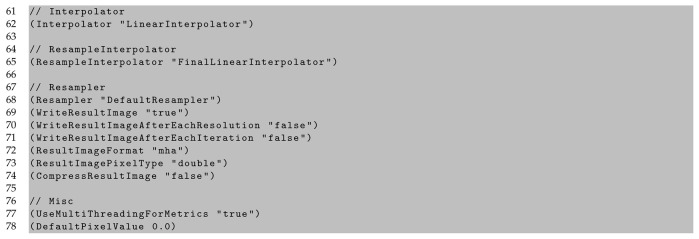
Elastix parameter file for rigid in vivo T1 to ex vivo T1 registration.

**Listing A2:**
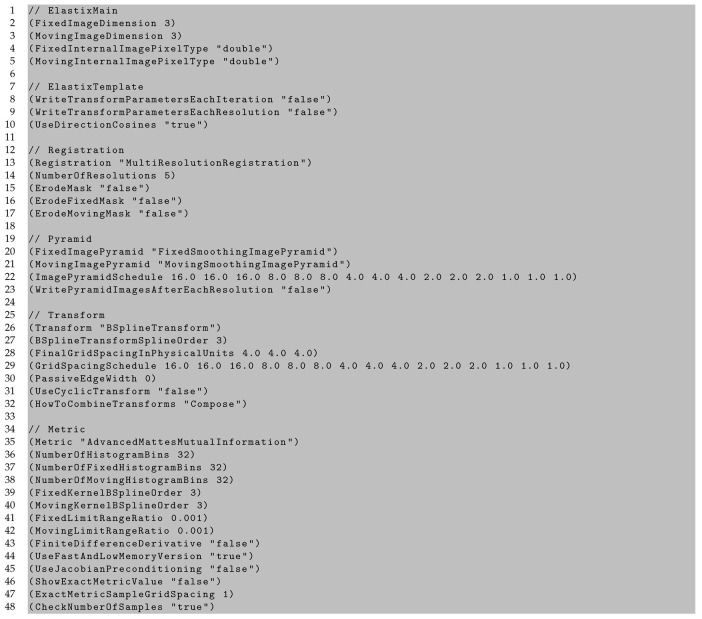

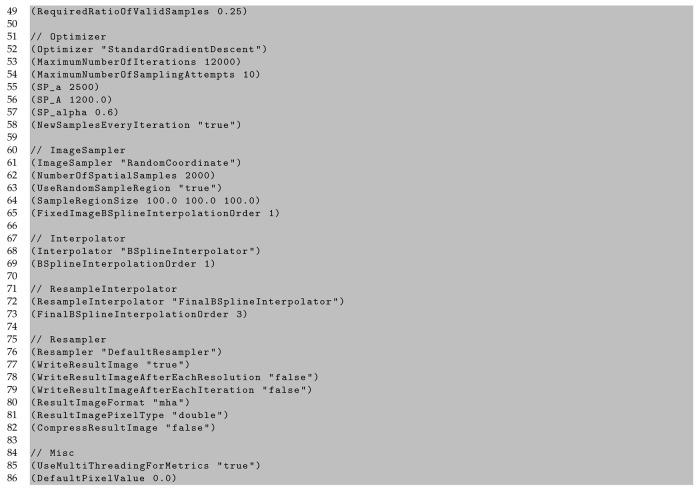
Elastix parameter file for B-spline in vivo T1 to ex vivo T1 registration.

**Figure A3:**
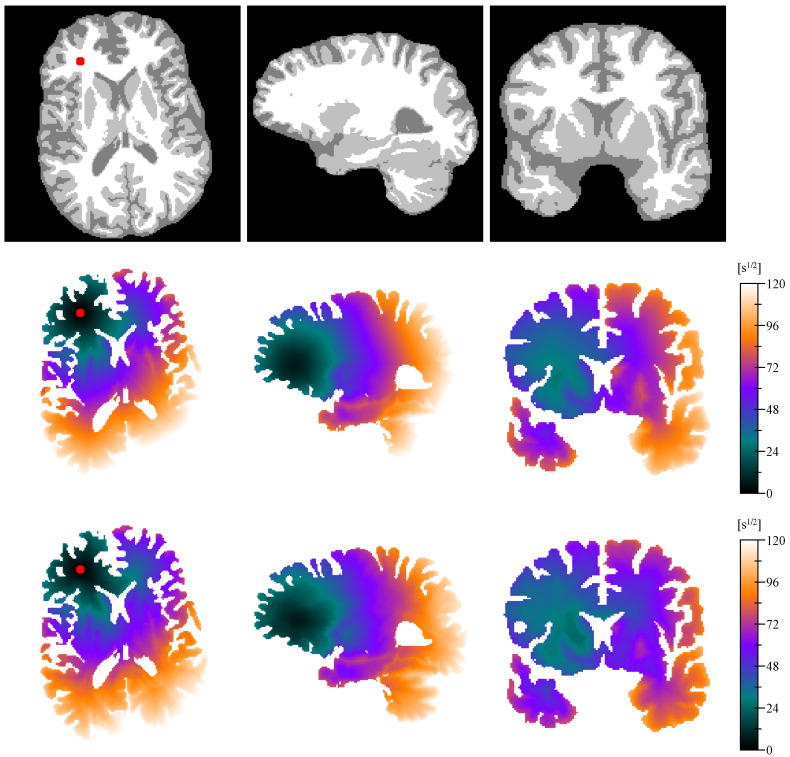
Segmented brain map (**first row**) and arrival time maps from a sphere with radius 2.5 mm (red dot) obtained using an adapted implementation of the anisotropic fast marching algorithm in [23] for an isotropic (**second row**) and a DTI-derived anisotropic (**third row**) metric tensor in a healthy volunteer.

## Data Availability

The data presented in this study are available on reasonable request from the corresponding author.

## Abbreviations

The following abbreviations are used in this manuscript:

ADC	Average diffusion coefficient
AFM	Anisotropic fast marching
ASSD	Average symmetric surface distance
BBB	Blood–brain barrier
DTI	Diffusion tensor imaging
DW	Diffusion-weighted
FA	Flip angle
GBM	Glioblastoma
GVP	Glomeruloid vascular proliferation
HE	Hematoxylin and eosin
IHC	Immunohistochemistry
MR(I)	Magnetic resonance (imaging)
PD	Proton density
PET	Positron emission tomography
PPN	Pseudo-palisading necrosis
ReLU	Rectified linear unit
Susp.	Suspected
T1Gd	T1-weighted sequence with injection of gadolinium-based contrast agent
T2-FLAIR	T2-weighted sequence with fluid-attenuated inversion recovery
TE	Echo time
TI	Inversion time
TMZ	Temozolomide
TR	Repetition time
VEGF	Vascular endothelial growth factor
WHO	World Health Organization

## Appendix A. Slicer Design

The in vivo T2-FLAIR image was first resampled to an isotropic voxel size of 0.5 mm×0.5 mm×0.5 mm. A brain mask was generated using the Otsu thresholding [51], followed by a morphological opening of radius 1, a largest connected component extraction, and a morphological closing of radius 15. The brain mask bounding box was then drawn on a binary image with the same spacing and was evenly extended on both sides in each spatial dimension, with final dimensions of 160.5 mm×190.5 mm×172 mm. The brain mask volume was subtracted from the box volume. Ten slits of 12mm×190.5mm×152mm spaced by 5.5 mm were carved into the box along the sagittal plane for brain slicing and the box was split in half along the axial plane at the level of the largest brain mask outline for brain insertion. Four cylindric fixations with radius 5.5 mm and height 9.5 mm were added to each corner of the upper part of the slicer and subtracted from its lower part. A 3D surface mesh was finally generated from the binary image using the marching cubes algorithm and exported in stl format for printing. The brain slicer design is depicted in Figure A1.

Ten cutting guides were also designed to ease the cutting of the brain slices into blocks. Those consisted of 190.5 mm×149.5 mm×11 mm plates from which the slice volume with a thickness of 5.5 mm was subtracted. Grooves were carved into the plates to guide the scalpel-cutting of 26 mm×18 mm×5.5 mm tissue blocks whose dimensions are compatible with the histological processing chain at our institution. All image processing and design steps were performed using an in-house software in C++ based on VTK [20] and ITK [21].

The brain slicer was printed using 1.75 mm PLA thread on a HICTOP 3DD-17-ATL-FM printer (HIC Technology, Shenzhen, China) with a nozzle size of 0.4 mm (layer thickness 0.2 mm, honeycomb filling 5%) for a total printing time of 96 h. The printing of the ten cutting guides was parallelized on 5 Prusa i3 MK2 printers (Prusa Research, Prague, Czech Republic) with equivalent settings for a printing time of around 10 h per guide.

## Appendix B. Deep Learning-Based Nuclei Counting

For each of the 28 stained slides resampled to a pixel size of 1 μm×1 μm, 50 tiles of 100 px×100 px were randomly chosen after exclusion of background tiles. Background tiles were defined as tiles containing less than 10% of tissue pixels, empirically chosen as pixels whose the lowest RGB value is above 220 for our calibrated scanner. The 1600-tile dataset was then weakly supervised using an in-house annotation program in Python allowing the user to locate each cell nucleus within a tile by a simple mouse click. For each annotated tile, a nuclei presence probability map was generated by the superposition of 2D Gaussian blobs centered on user-pointed nucleus locations with full width at half maximum of 3 px (i.e., standard deviation σ≈1.27px), truncated to a radius of 4σ. The supervised dataset was split into training and validation sets in proportion 80–20%.

A U-Net [52] deep convolutional neural network was implemented for cell nuclei detection, consisting of two downsampling blocks, three upsampling blocks and an output block. Each downsampling block is made of two convolutional layers with kernel size 3×3 and stride 1 followed by a bias-adding layer and a rectified linear unit (ReLU) activation layer. A max pooling layer with kernel size 2×2 and stride 2 is added at the end of the block to reduce the feature maps dimensions by a factor 2. The upsampling blocks are identical to the downsampling blocks except that the max pooling layer is replaced by a deconvolution layer with kernel size 2×2 and stride 2 followed by a bias-adding layer and a ReLU activation layer to expand the feature maps dimensions by a factor 2. Skip connections are added between the output of the second convolutional layer of each downsampling block and the input of the corresponding upsampling block with the same spatial dimensions, implemented as a concatenation operation. The output block has the same structure as the downsampling blocks except that the max pooling layer is replaced by a convolutional layer with kernel size 1×1 and stride 1 followed by a bias-adding layer and a sigmoid activation layer to merge the last 128 feature maps into a single presence probability map. The network architecture is depicted in Figure A2 and was implemented in Python using the TensorFlow framework (version 1.13.1) [53].

The network was trained on the weakly-supervised training set using the cross-entropy loss and the Adam optimizer [54] (learning rate: 10^−4^, $\beta_{1}$: 0.9, $\beta_{2}$: 0.999, ϵ: 10^−6^). An evaluation was performed on the validation set at the end of each epoch and the training was stopped early after no improvement of the validation metric in 100 epochs. The network parameter values that gave the best validation metric value (0.041) were kept, which was reached at epoch 96. A similar weakly supervised deep learning approach was used in [55] for cell nuclei detection in histological slides, but Euclidian distance to user-pointed nuclei locations and the mean squared error loss were used instead of Gaussian nuclei presence probability maps and the cross-entropy loss.

After training, each resampled scanned slide was divided into adjacent tiles of 100 px×100 px from which nuclei presence probability maps were predicted by the trained network. A nuclei count was finally derived for each predicted probability map by detecting its local maxima with a value greater or equal to 0.1 and spaced by at least 3 px.

## Appendix C. In Vivo to Ex Vivo Registration

Registration of the in vivo T2-FLAIR image to the ex vivo T1 image was performed using the Elastix software (version 4.801) [22] as described in Section 2.7. The Elastix parameter files for rigid and B-spline registration are, respectively, given in Listing A1 and Listing A2.

## Appendix D. Pathological Results

The results of the pathological examination and numerical tile processing are summarized in Table A1.

**Table A1:** Results of the pathological examination and numerical tile processing. PPN: pseudo-palisading necrosis, GVP: glomeruloid vascular proliferation, susp.: suspected.

					Cell Density [$10^{3}$ cell m$m^{-2}$]			Distance [mm]		
**Block**	**PPN**	**Tumor Cells**	**GVP**	**Edema**	**Min**	**Max**	**Mean**	**Min**	**Max**	**Mean**
1	No	No	No	No	1.08	20.46	11.77	33.40	40.40	34.95
2	No	No	No	No	2.25	23.88	13.80	35.05	46.76	40.12
3	No	Infiltrative (susp.)	No	Yes	1.52	25.25	14.96	7.24	21.18	14.00
4	No	No	No	No	1.36	20.64	12.50	29.36	41.66	36.84
5	No	No	No	Yes	1.19	24.85	15.40	11.74	30.27	21.57
6	No	Infiltrative (susp.)	No	No	3.72	28.44	18.70	32.92	40.35	36.52
7	No	Infiltrative (susp.)	No	Yes	0.89	38.95	21.44	38.59	61.70	49.04
8	Yes	Block	Yes	No	3.37	37.29	23.65	0.50	5.17	2.71
9	Yes	Infiltrative	Yes	Yes	1.02	44.37	31.71	0.50	4.72	1.91
10	No	Infiltrative (susp.)	No	Yes	5.52	37.15	25.53	0.50	3.71	1.06
11	No	No	No	No	1.73	25.68	15.21	19.85	38.74	30.74
12	No	Infiltrative	Yes	Yes	0.89	36.08	19.70	0.50	31.46	14.24
13	Yes	Block	Yes	No	1.40	52.46	21.69	0.50	22.85	9.72
14	No	No	No	No	1.10	19.25	11.78	17.73	32.20	25.51
15	No	No	No	Yes	2.08	22.90	11.41	28.63	54.74	40.14
16	No	No	No	No	0.99	26.47	13.52	20.37	48.79	35.91
17	No	No	No	No	1.42	23.69	13.10	28.30	56.12	42.88
18	No	No	Yes	Yes	2.46	39.29	20.71	7.94	27.01	16.83
19	Yes	Block	Yes	Yes	0.99	44.12	19.82	0.50	18.50	6.92
20	Yes	Block	Yes	No	6.25	61.05	28.40	0.50	7.37	2.06
21	No	Infiltrative	Yes	Yes	4.75	34.20	23.05	0.50	14.62	7.70
22	No	No	No	Yes	0.86	30.37	10.79	2.99	36.87	21.39
23	No	No	No	No	0.94	25.61	13.23	21.14	49.61	34.48
24	No	No	No	No	1.37	26.72	13.72	57.27	90.93	71.08
25	No	Infiltrative (susp.)	No	Yes	0.87	39.07	21.03	1.71	21.41	10.87
26	Yes	Block	Yes	Yes	6.21	54.24	22.79	0.50	8.81	2.96
27	No	No	No	Yes	0.93	37.96	21.33	12.74	30.35	18.40
28	No	No	No	No	0.95	34.79	20.76	14.35	35.85	22.57

## Appendix E. Isotropic vs. Anisotropic Metric Tensor

To assess the impact of an anisotropic metric tensor on the computed arrival time maps, T1 BRAVO (TR = 8.332 ms, TE = 3.088 ms, TI = 450 ms, FA = 12${}^{{}^{\circ}}$, pixel bandwidth = 244 Hz ${\mathrm{px}}^{-1}$, slice thickness/spacing = 1/1 mm, matrix = 240 px×240 px) and EPI DTI (TR = 7000 ms, TE = 77.1 ms, TI = 108 ms, FA = 90${}^{{}^{\circ}}$, pixel bandwidth = 1953.12 Hz ${\mathrm{px}}^{-1}$, phase/slice acceleration factor = 2/1, multiband factor = 3, slice thickness/spacing = 2/2 mm, matrix = 120 px×120 px, directions = 32, b-value = 1000 s m$m^{-2}$) MR acquisitions of a healthy volunteer were performed on a 3T Signa PET/MR scanner (GE Healthcare, Chicago, IL, USA). The brain domain was extracted on the T1 image resampled to 1 mm×1 mm×1 mm, then segmented into white matter, gray matter, and cerebrospinal fluid using the MICO algorithm [56]. The DTI data were corrected for motion, eddy currents, and susceptibility artifacts using the ‘eddy’ and ‘topup’ tools and a water diffusion tensor field was reconstructed using the ‘dtifit’ tool, all these tools being available as part of the FSL software [57]. A unit tumor diffusion tensor field was derived from the segmented brain map and the DTI-derived water diffusion tensor field using the method proposed by Jbabdi and colleagues in [14] with a white matter/gray matter diffusivity ratio of 10 and an anisotropy factor of 10, then resampled to 1 mm×1 mm×1 mm. By unit tensor, it is meant that the tensor mean diffusivity $\mathrm{MD}=\mathrm{Tr}\left(D\right)/3$ is equal to 1 at every white matter voxel. Arrival time maps from a sphere with radius 2.5 mm located in the right frontal lobe—similarly to the GBM case studied—were finally computed using an adapted implementation of the AFM algorithm proposed in [23] with the inverse tumor diffusion tensor used as metric [27]. The results are depicted in Figure A3.

The results suggest even shorter arrival times in the contralateral hemisphere in the anisotropic case due to the high anisotropy in the corpus callosum region, which is not in favor of a coincidence between the edema outlines and a thresholded arrival time map contour (see Figure 7).
